# Potential Antinociceptive Effects of Chinese Propolis and Identification on Its Active Compounds

**DOI:** 10.1155/2018/5429543

**Published:** 2018-09-26

**Authors:** Liping Sun, Lei Liao, Bei Wang

**Affiliations:** ^1^Institute of Apicultural Research, Chinese Academy of Agricultural Sciences, Beijing 100093, China; ^2^Institute of Clinical Pharmacy of Beijing Municipal Health Bureau, Beijing 100035, China; ^3^College of Bee Science, Fujian Agriculture and Forestry University, Fuzhou 350002, China

## Abstract

Propolis is an important bee product which has been applied to the treatment of several diseases. The aim of this study was to understand the material basis of Chinese propolis on pain relief; different Chinese propolis fractions (40W, 40E, 70E, and 95E raw propolis extracted followed by 40%, 70%, or 95% ethanol) were prepared, and their antinociceptive effects were evaluated. By analyzing using UPLC-Q-TOF-MS, we showed that 40W was rich in phenolic acids, like caffeic acid, while 40E, 70E, and 95E have relatively high levels in flavonoids, like galangin, pinocembrin, and chrysin. Notably, chrysin amounts in 70E and 95E are much higher than those in 40E fraction. Antinociceptive effects by these propolis fractions were evaluated in mice using acetic acid-induced writhing test, hot plate test, and tail immersion test, respectively. We noticed that only 40E fraction showed a significant reduction on acetic acid-induced writhing test. Importantly, in the hot plate test, all groups showed their effectiveness, except for the 70E group. We also noticed that 40W, 40E, and 95E administration caused an increase in the tail withdrawal latency of the mice. These data suggested that the different antinociceptive effects of different fractions from Chinese propolis extracts are directly link to their flavonoid composition.

## 1. Introduction

The inflammation displays with classical symptoms of pain, swelling, and heat. Pains happened in the freakishness of any part of the body, including spine joints, tendons, muscles, internal organs, and ligaments. Relief of pain is required for the quality and normal life activities [1]. Inflammation is the genesis of pains, and recent findings suggested that inflammatory cytokines provide necessary evidence for alleviating pains during inflammation. Despite the modern pain relief drugs (most of them are nonsteroidal anti-inflammatory drugs (NSAID)) that are grossly used based on these basic concepts, significant adverse effects happened, including addiction, gastric lesions, tolerance, and sedation [2]. Therefore, new therapeutic strategies for the treatment/relief for pains with less side effects are warranted.

The usage of herbal medicine in the treatment for various kinds of diseases has a long history [3]. Modern preclinical studies have been applied for testing the effectiveness of the herbal medicines for relieving pains using various models, including nociception test against mechanical, thermal, and chemical stimuli, or using rodent inflammatory pain models [4, 5]. The antinociceptive effects of the herbal medicines might attribute to their anti-inflammatory effects, which interact with the inflammatory-associated enzymes (like COX-1/COX-2), or regulate the cytokine production.

Propolis is an important hive product collected by the honeybees (*Apis mellifera*) from various botanic sources (like tree buds), which has received great interest in medicine due to its versatile pharmacological activities, including antioxidant [6], antinociceptive [7], and anti-inflammatory [8]. It has been shown that the chemical compositions of propolis from different geographical regions of origins are dissimilar, depending on their botanic sources. We demonstrated previously that the anti-inflammatory effect of Chinese propolis, which is mainly originated from poplar trees, is associated with the polyphenolic constitutions, including flavonoids (like pinocembrin, chrysin, and quercetin) as well as phenolic acids and its esters (like caffeic acid and caffeic acid phenethyl ester (CAPE)) [9]. Despite the mechanisms of the anti-inflammatory action beyond these active constitutions that are still not fully understood in CP, it is still recognized that the this action can be attributed to its direct modulating effects on the inflammatory cytokine releases as well as inflammatory signaling pathways, like NF-*κ*B, and free radical scavenging properties on ROS [10].

It has been recognized as a critical issue for understanding the material basis of herbal medicine, which is necessary for the modern medicine development as well as clinical applications. Here, we used different Chinese propolis fractions and several in vivo animal models were applied to provide systemic data for elucidating the potential antinociceptive effects of Chinese propolis.

## 2. Results and Discussion

### 2.1. Phytochemical Analysis and *In Vitro* Antioxidant Activities of Different Fractions of CP

The abundant polyphenolic compounds are known as key contributors to the therapeutic effects of propolis. It has been known that different solvents will affect the yield of these bioactive constituents. We first performed the phytochemical analysis on the bioactive fractions of CP (40W, 40E, 75E, and 95E). As shown in Table 1, 40E fraction showed the highest TPC and TFC values, while 40W is the lowest among the 4 groups. Based on previous published studies, the total flavonoid content in CP ranged from 42.9 to 302 mg GAE/g. Accordingly, the total phenolic acid content of CP varied from 8.3 to 188 mg QE/g. Hence, the present study reported values of CP fall into these ranges [11].

DPPH free radical scavenging activity assay and FRAP assay are widely used in the screening of natural antioxidant as well as plant extracts. Previous studies have been performed exhaustively on the free radical scavenging of propolis [11], while the antioxidant potential among our four CP fractions in the present study is quite different. The 75E group showed the best DPPH-scavenging activity and reducing power, which correlated well with its high TPC and TFC values. Interestingly, the 40W fraction is the second best on the *in vitro* antioxidant assay, despite its relatively low values in the TPC and TFC.

**Figure 1:**
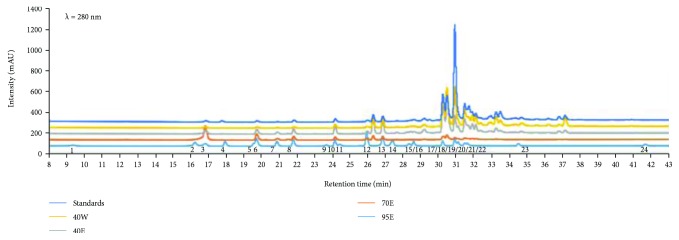
Base peak chromatogram in the UV spectrum in the 280 nm of the extract of different fractions on Chinese propolis.

Polyphenolic constitutions, including phenolic acids and flavonoids, are regarded as major contributors to the antioxidant activities. Generally, our results showed to be in parallel with other studies in which all CP fractions have potent antioxidant properties. However, we noticed that the 40W group showed the best *in vitro* free radical scavenging activities, suggested that this fraction contained rich nonpolar phenolic contents. Those phenolic acids contain aromatic rings which have one/more hydroxyl groups and enable quenching free radicals by forming resonance-stabilized phenoxyl radicals [12]. As shown in Table 2 and Figure 1, we also analyzed 20 phenolic compounds in CP using HPLC-DAD/Q-TOF-MS.

### 2.2. Effects of CP Administration on Acetic Acid-Induced Writhing Test

**Figure 2:**
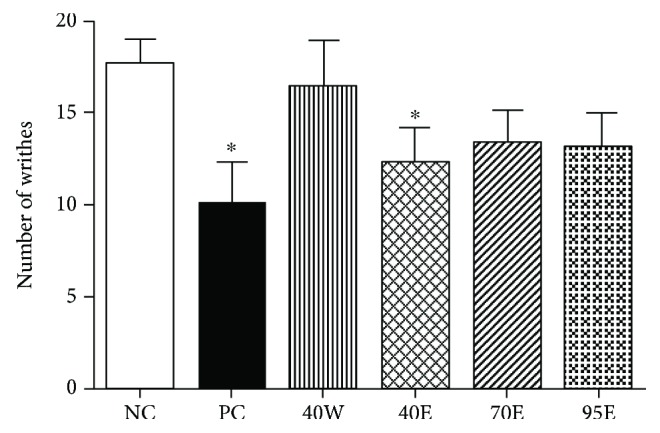
Effects of oral administration of different fractions of Chinese propolis extracts on acetic acid-induced writhing test on acetic acid-induced visceral nociception in mice (*n* = 8 per group). ^∗^*p* < 0.05 versus the control group. The values are expressed as means ± SD.

Rodent writhing test model induced by the acetic acid is a typical study for antinociceptive studies. This model has high sensibility for the screening of a number of diseases, like nonsteroidal anti-inflammatory drugs (NSAIDs), muscle relaxant, and depressant drugs [13]. The writhing test model is also known as the abdominal constriction response, which enables the detection the antinociceptive effects and dose levels of the drugs. In our study, we first noticed that only the 40E fraction at 5 g/kg p.o. showed a significant reduction (*p* < 0.05), as compared with the control group. The 40E group's effect (5 g/kg p.o.) was also similar to that of ibuprofen (60 mg/kg, positive control) (Figure 2). Previous studies used the black Moroccan propolis water extract of propolis and did the writhing test in rats, and they noticed that the maximum percentage inhibition of constrictions of 49% was observed at 5% for the extract [14]. It should be noticed that the vegetation and major chemical profiles of Moroccan propolis are different from the poplar-type Chinese propolis samples we used in the present study. The major botanic source of that propolis is *Ceratonia siliqua* (Fabaceae) and *Pistacia lentiscus* (Anacardiaceae) [15]. These data further support the effectiveness of propolis from different geographic regions with varied chemical composition.

### 2.3. Effect of CP Administration on Hindpaw Lick Latency of the Mice under Hot Plate Test

**Figure 3:**
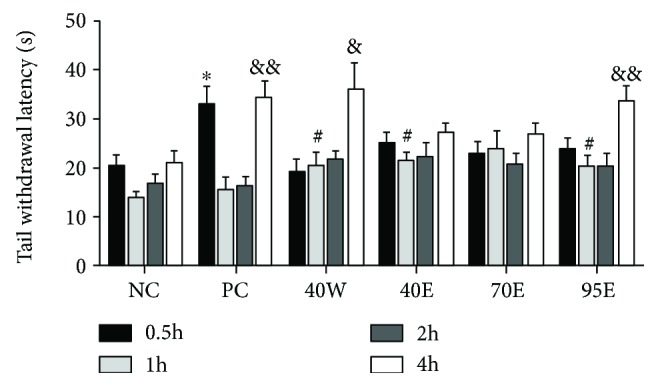
Effects of oral administration of different fractions of Chinese propolis extracts on hindpaw lick latency of the mice under hot plate test (*n* = 8 per group). ^∗^*p* < 0.05 versus the NC group at 0.5 h; ^#^*p* < 0.05 versus the NC group at 1 h and ^&^*p* < 0.05 and ^&&^*p* < 0.01 versus the NC group at 3 h. The values are expressed as means ± SD.

For assessing the opioidergic analgesic mechanisms as well as narcotic analgesia, the hot plate test is a classical model [16]. As shown in Figure 3, similar to the positive control drug (ibuprofen, 60 mg/kg), we noticed that the 40W and 95E groups were able to boost the tail withdrawal latency and these effects were influenced independently by the mice's licking response of the pain threshold. Nevertheless, the effectiveness of propolis extracts against the hot plate test remains controversial. Bulgarian propolis and Brazilian propolis were previously tested using this model but the results were ineffective [17]. Different black Moroccan propolis (water extracts) caused a marked analgesic effect using the hot plate test. It could be suggested that some active constitutes with analgesic effect are presented in the water extract of Moroccan propolis.

### 2.4. Effect of CP Administration on Tail Withdrawal Latency of the Mice under Tail Immersion Test

**Figure 4:**
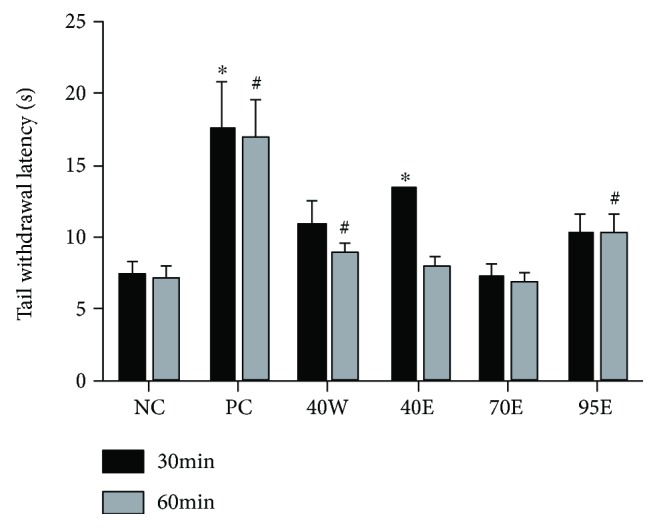
Effects of oral administration of different fractions of Chinese propolis extracts on tail withdrawal latency of the mice under tail immersion test in mice (*n* = 8 per group). ^∗^*p* < 0.05 versus the NC group at 0.5 h; ^#^*p* < 0.05 versus the NC group at 1 h. The values are expressed as means ± SD.

The antinociceptive effects of CP fractions as well as ibuprofen (60 mg/kg) were tested by the use of a thermal nociceptive stimulation (tail immersion in a 48°C water bath). As shown in Figure 4, all test groups, except for the 70E group, caused an increase in the tail withdrawal latency of animals administrated with them. It is a variant of the tail-flick pain model and is a sensitive and particularly useful test for demonstrating dose-related activity [16]. The effectiveness of analgesics in this model is also highly correlated with relief of human pain. CP fractions significantly attenuated thermal nociception in rats in this model, though not as effectively as ibuprofen. The tail immersion test gives a response that is believed to be a spinally mediated reflex but the mechanism of response could also involve higher neural structures. It is therefore suggested that CP exerts its antinociceptive effects, at least in part, by spinally mediated central mechanisms.

## 3. Discussion

In summary, our data suggested that different fractions from Chinese propolis extracts enriched in polyphenolic constitutions showed central and peripheral antinociceptive effects that can be linked with their antioxidant activities. These results support the clinical usage of propolis as an alternative approach for painful disease treatment. Since the antinociceptive properties are closely linked to the anti-inflammatory effects, which we have not fully understood, the modulating effect of these fractions on the inflammatory cytokine releases is still needed to be explored in the future.

## 4. Materials and Methods

### 4.1. Reagents

Acetic acid, DPPH, and ABTS, as well as standards for the chemical analysis, were obtained from Sigma-Aldrich (St. Louis, Mo., USA). Methanol was obtained from Fisher Scientific (Pittsburgh, PA, USA). All other reagents were obtained from Sangon Biotechnology (Shanghai, China) or as indicated in the specified methods.

### 4.2. Propolis Collection, Extraction, Active Compound Separation, and Determination

Chinese propolis (CP), which was originated from poplar (*Populus* sp.) was collected from our apiary in Shandong Province, China. A voucher specimen was deposited at the Institute of Apicultural Research, Chinese Academy of Agricultural Sciences, China. As described in the previous study, the propolis were extracted using 40% ethanol for the first time at 30°C, under vacuum. Afterwards, the propolis residues were collected and dried to get 40% ethanol upper fraction (40W) and the supernatants were dried to get 40% propolis extracts (40E). Then, 70% and 95% ethanol residues were extracted subsequently, to obtain 70% ethanol extracted propolis (70E) and 95% ethanol extracted propolis (95E), respectively [18]. Propolis total phenolic contents were determined by the Folin-Ciocalteu method and showed as milligram (mg) gallic acid equivalents (GAE)/g. The aluminum chloride colorimetric method was applied to measure total flavonoid contents and showed as mg quercetin equivalents (QE)/g [19]. *In vitro* free radical scavenging activity was performed by the DPPH assay and ferric reducing antioxidant power (FRAP), respectively [20]. Polyphenolic extracts of propolis were analyzed using HPLC-DAD/Q-TOF-MS on an Agilent 1200 series rapid resolution LC system coupled with Agilent 6510 ESI-Q-TOF (Agilent Technologies, CA, USA). A gradient elution was operated using a mobile phase A, 0.05% formic acid water, and mobile phase B, methanol. The gradient program was 1% (B) at 0–2 min, 1–20% (B) at 2–10 min, 20–45% (B) at 10–18 min, 45–82% (B) at 18–28 min, 82–95% (B) at 28–40 min, 95–95% (B) at 40–50 min, 95–100% (B) at 50–55 min, and 100% (B) at 55–70 min, with the flow rate of 0.25 ml/min. The injection volume was 1 *μ*l, and the UV spectra were detected ranging from 190–400 nm with DAD detector; the chromatograms were recorded at 210, 254, 280, 320, and 360 nm.

### 4.3. Antinociceptive Tests

#### 4.3.1. Animals

Male ICR mice (20 ± 2 g) were purchased from the Vital River Laboratory Animal Technology Co. Ltd. (Beijing, China). The mice were kept under controlled standard environment conditions. All experimental protocols were approved by the Animal Ethical Committee of Institute of Apicultural Research, CAAS.

#### 4.3.2. Acetic Acid-Induced Writhing Test

The 0.85% acetic acid was intraperitoneally injected to the mice to induce peritoneal irritation, with typical symptoms of abdominal contortions as well as hind limb extensions [13, 14]. The mice were randomly divided into 6 groups (*n* = 8): vehicle control, standard drug (ibuprofen 60 mg/kg), and 5 g/kg bioactive fractions of CP (40W, 40E, 75E, and 95E) for 8 d. Propolis were administrated to the mice by gavage every day. On the 8^th^ day, the mice were injected with acetic acid (0.1 ml/10 g). Then, the mice were moved to polyethylene boxes and the abdominal contortions were observed and recorded. Nociceptive behaviors were quantified 15 minutes after the acetic acid injection.

#### 4.3.3. Hot Plate Test

The hot plate analgesia meter (Ugo Basil, Italy; Socrel DS-37) was used for generating a heated surface (55 ± 0.2°C). The mice were moved to the glass cylinder with a diameter of 20 cm and put on the heated surface. Treated mice received oral vehicle, drug standard (ibuprofen 60 mg/kg, GSK China), and 5 g/kg bioactive fractions of CP (40W, 40E, 75E, and 95E) for 7 d. On the 7th day, the latency to nociceptive response of the mice was recorded at 0.5, 1, 2, and 4 h after oral treatment with different samples. Paw licking and jumping were evaluated as the indices of the thermal reactions.

#### 4.3.4. Tail Immersion Test

The tail immersion test involved immersing the extreme 4 cm of the mouse tail in a water bath containing water at a temperature of 45 ± 0.5°C [21]. The mice react by withdrawing the tail. The reaction time was recorded with a stopwatch. The mice were randomly selected to perform in one of the study groups (five per group): control, ibuprofen (60 mg/kg), and 5 g/kg bioactive fractions of CP (40W, 40E, 75E, and 95E). The reaction time (Ta) for the study groups was taken at a latency period of 30 min and 1 h following the administration of the drugs or extract.

### 4.4. Statistical Analysis

Data are expressed as the means ± SD for the indicated number of independently performed experiments. Statistical comparison of the data was performed by using Student's *t* test or one-way ANOVA using the Student–Newman–Keules method. *p* values less than 0.05 were considered statistically significant. All statistical tests were carried out using SPSS 17.0.

## Figures and Tables

**Table 1:** Total phenolic content (TPC), total flavonoid content (TFC), reducing power, and DPPH free radical scavenging activities of different fractions of CP.

	TPC (mg GAE/g)	TFC (mg QE/g)	Reducing power (*μ*g BHT/ml)	DPPH-scavenging activity IC50 (*μ*g BHT/ml)
BHT	/	/	/	82.42 ± 3.7
40W	175.5 ± 0.8	9.3 ± 0.1	118.7 ± 7.8	27.7 ± 1.2
40E	515.8 ± 4.0	142.7 ± 0.6	128.6 ± 3.9	19.5 ± 2.0
75E	270.5 ± 28.9	125.9 ± 18.0	87.1 ± 2.1	52.5 ± 3.5
95E	280.9 ± 0.3	126.9 ± 3.4	68.2 ± 4.0	78.8 ± 6.2

^a^Values are the means ± SD (*n* ± 3).

**Table 2:** HPLC-DAD/Q-TOF-MS analysis on Chinese propolis.

Peak	Compounds	RT (min)	(*M* + 1)+	Content (mg/g)			
				40E	40W	70E	95E
1	Protocatechuic acid	9.525	155.0339	/	/	/	/
2	Vanillic acid	16.445	169.0495	/	/	/	/
3	Caffeic acid	16.996	181.0495	7.24	44.03	1.84	1.66
4	Syringic acid	18.13	199.0601	/	/	/	/
5	7-Hydroxycoumarin	19.751	163.0389	/	/	/	/
6	P-Coumaric acid	19.929	165.0546	7.59	15.62	1.77	1.41
7	Ferulic acid	21.063	195.0652	3.90	5.71	0.76	0.53
8	Isoferulic acid	22.003	195.0652	7.79	10.28	1.43	1.97
9	Rutin	23.85	611.1607	/	/	/	/
10	3,4-Dimethoxycinnamic acid	24.337	209.0808	17.20	8.53	4.78	3.62
11	Myricetin	24.839	319.0448	/	/	/	/
12	Trans-cinnamic	26.135	149.0597	/	/	/	/
13	Quercetin	26.865	303.0499	0.09	/	0.03	0.05
14	Pinobanksin	27.043	273.0757	12.87	4.18	6.69	2.99
15	Luteolin	27.529	287.055	0.76	0.23	0.23	0.18
16	Kaempferol	28.501	287.055	4.77	1.14	2.03	1.51
17	Apigenin	28.793	271.0601	9.18	2.10	2.02	1.06
18	Pinocembrin	30.43	257.0808	9.30	9.40	34.34	27.52
19	Chrysin	31.11	255.0652	24.37	4.85	37.66	55.03
20	CAPE	31.305	285.1121	10.65	2.68	5.28	2.15
21	Galangin	31.661	271.0601	37.77	10.62	39.00	31.06
22	Curcumin	31.823	369.1333	/	/	/	/
23	Artepillin C	34.675	301.1798	/	/	/	/
24	*α*-Mangostin	41.854	411.1802	/	/	/	/

mg/g means the content of compounds per g extract of different fractions.

## Data Availability

The data used to support the findings of this study are available from the corresponding author upon request.
